# Effect of polyethelene oxide on the thermal degradation of cellulose biofilm – Low cost material for soft tissue repair in dentistry

**DOI:** 10.4317/jced.53465

**Published:** 2017-07-01

**Authors:** Anna Akkus, Rakim Tyler, David Schiraldi, Renato Roperto, Fady Faddoul, Sorin Teich

**Affiliations:** 1 School of Dental Medicine, Department of Comprehensive Care, Case Western Reserve University, Cleveland, Ohio 44106, USA; 2 School of Engineering, Department of Macromolecular Science, Case Western Reserve University, Cleveland, Ohio 44106, USA

## Abstract

**Background:**

Bio cellulose is a byproduct of sweet tea fermentation known as kombusha. During the biosynthesis by bacteria cellulose chains are polymerized by enzyme from activated glucose. The single chains are then extruded through the bacterial cell wall. Interestingly, a potential of the Kombucha’s byproduct bio cellulose (BC) as biomaterial had come into focus only in the past few decades. The unique physical and mechanical properties such as high purity, an ultrafine and highly crystalline network structure, a superior mechanical strength, flexibility, pronounced permeability to gases and liquids, and an excellent compatibility with living tissue that reinforced by biodegradability, biocompatibility, large swelling ratios.

**Material and Methods:**

The bio-cellulose film specimens were provided by the R.P Dressel dental materials laboratory, Department of Comprehensive Care, School of Dental Medicine, Case Western Reserve University, Cleveland, US. The films were harvested, washed with water and dried at room temperature overnight. 1wt% of PEG-2000 and 10wt% of NaOH were added into ultrapure water to prepare PEG/NaOH solution. Then bio-cellulose film was added to the mixture and swell for 3 h at room temperature. All bio-cellulose film specimens were all used in the TA Instruments Q500 Thermogravmetric Analyzer to investigate weight percent lost and degradation. The TGA was under ambient air conditions at a heating rate of 10ºC/min.

**Results and Conclusions:**

PEG control exhibited one transition with the peak at 380ºC. Cellulose and cellulose/ PEG films showed 3 major transitions. Interestingly, the cellulose/PEG film showed slightly elevated temperatures when compared to the corresponding transitions for cellulose control. The thermal gravimetric analysis (TGA) degradation curves were analyzed. Cellulose control film exhibited two zero order transitions, that indicate the independence of the rate of degradation from the amount on the initial substance. The activation energies for three transitions for cellulose and cellulose/PEG showed increasingly higher values for the transitions at higher temperatures.

** Key words:**TGA, Bio-cellulose, PEG.

## Introduction

Bio cellulose (BC) is a byproduct of sweet tea fermentation known as Kombucha (1). The bacterial cellulose is secretion product of vinegar bacteria that is pure cellulose; BC has the same chemical structure as plant based cellulose however without lignin or any other substances. During the biosynthesis by bacteria cellulose chains are polymerized by CesA enzyme from activated glucose. The single chains are then extruded through the bacterial cell wall (2). The secreted macromolecules subsequently assemble into micro fibrils (3,4) and finally into bundles. The loosely assemble bundles then form cellulose ribbons that are organized as porous 3D networks that are stabilized via inter- and intra- hydrogen bonding (5,6).

Interestingly, a potential of the Kombucha’s byproduct bio cellulose (BC) as biomaterial had come into focus only in the past few decades (7). That is why among vast array of advanced biomaterials investigated for various tissue engineering applications, biol cellulose (BC) has not been extensively evaluated (8) despite of the material’s strong potential to become a high value product in the field of dentistry.

The unique physical and mechanical properties such as high purity, an ultrafine and highly crystalline network structure, a superior mechanical strength, flexibility, pronounced permeability to gases and liquids, and an excellent compatibility with living tissue that reinforced by biodegradability, biocompatibility, large swelling ratios (9,10). BC is defined by a wide range of applica-tions from optically functional materials (11), composites (12), and novel materials for medical applications such as wound dressing (13,14). However, there are no reports addressing potential fabrication and bio viability of the kombucha generated BC for dental soft tissue repair.

In this work we have examined the effect of polyethylene glycol (PEG) incorporation on degradation behavior of bio-cellulose, which would be indicative of bio cellulose specimen stability and potential to form soft, “skin” like feel upon touch that is attribu-ted to moisture absorbent materials.

## Material and Methods

The bio-cellulose film specimens were provided by the R.P Dressel dental materials laboratory, Department of Comprehensive Care, School of Dental Medicine, Case Western Reserve University, Cleveland, US. Glucose was purchased from Sigma-Aldrich (st. Louis, MO, US). Bio-cellulose film specimens (a symbiotic colony of Acetobacter and Saccharomyces) were cultured in deionized water with the following glucose concentration 5%. The films were harvested, washed with water and dried at room temperature overnight. Subsequently, bio- cellulose films was dried in vacuum at 350C overnight before PEG treatment. 1wt% of PEG-2000 and 10wt% of NaOH were added into ultrapure water to prepare PEG/NaOH solution. Then bio-cellulose film was added to the mixture and swell for 3 h at room temperature. Then the suspension was cooled down to -15ºC and held at that temperature overnight (12 h) until it became a solid frozen mass. All bio-cellulose film specimens were all used in the TA Instruments Q500 thermogravimetric analyzer to investigate weight percent lost and degradation. The TGA was under ambient air conditions at a heating rate of 10ºC/min from 25ºC - 800ºC.

## Results

PEG control exhibited one transition with the peak at 380 C0. Cellulose and cellulose/ PEG films showed 3 major transitions  Table 1. The cellulose/PEG film indicated slightly elevated temperatures when compared to the corresponding transitions for cellulose control, which is probably due to hydrogen bonding within the matrix that is introduced by the presence of PEG.

**Table 1:**
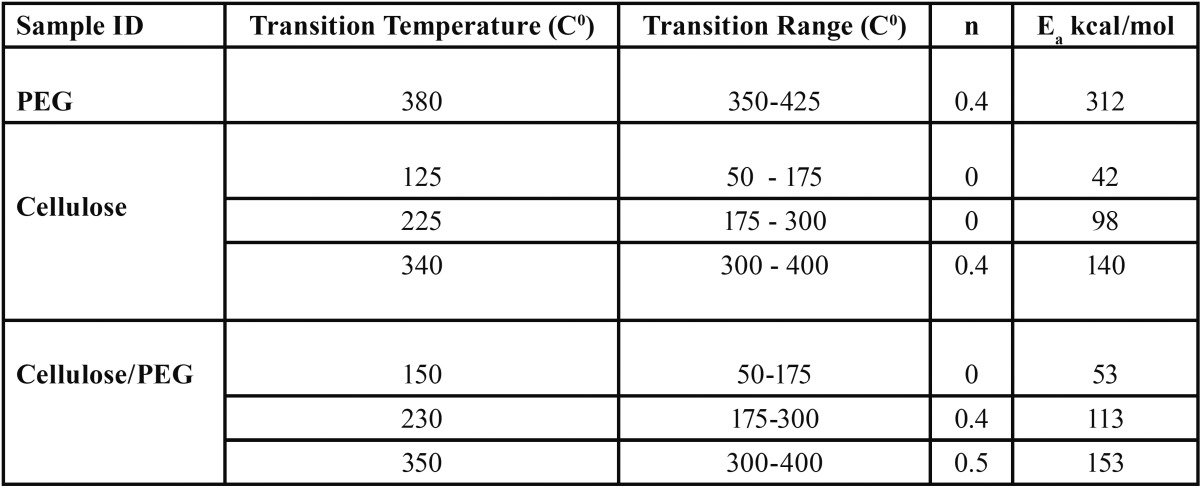
Thermal degradation measurements of the cellulose specimens and controls.

**Figure 1:**
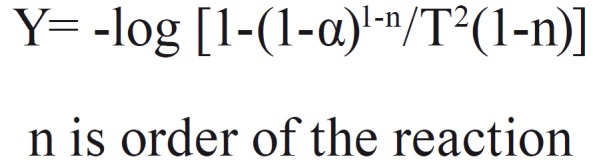
Equation.

**Figure 2:**
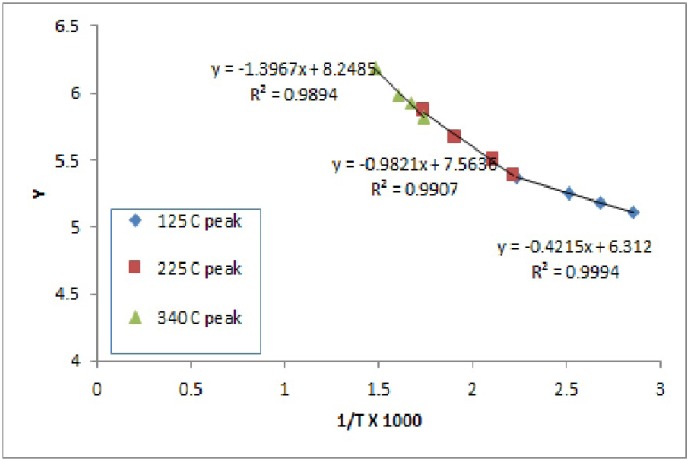
Analysis of cellulose degradation kinetics based on TGA measurement.

**Figure 3:**
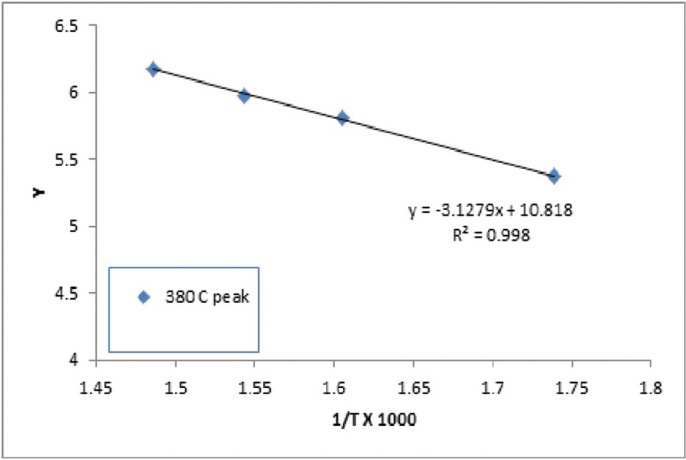
Analysis of PEG degradation kinetics based on TGA measurement.

**Figure 4:**
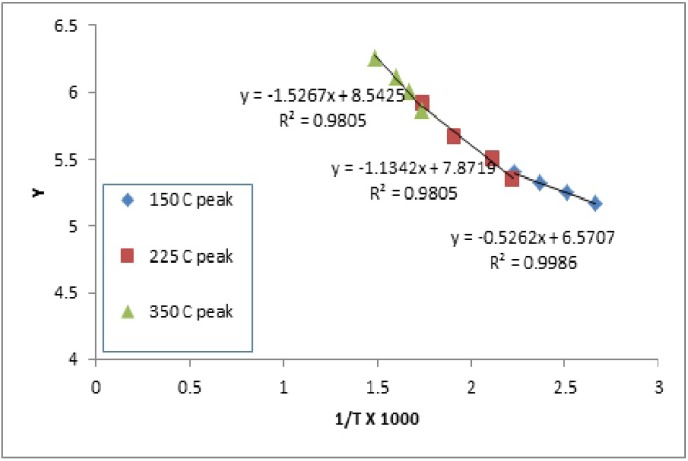
Analysis of PEG/cellulose degradation kinetics based on TGA measurement.

The thermal gravimetric analysis (TGA) degradation curves were analyzed. The kinetic parameters were derived from the thermogravimetric data (15,16). Cellulose degradation has exhibited three distinct regions, where, (Fig. 1):

The n value was chosen based on the best straight line through the points - on the assumption that the order is constant throughout the reaction. Cellulose control film exhibited two zero order transitions, that indicate the independence of the rate of degradation from the amount on the initial substance. This is usually indicative of enzymatically controlled processes. Interestingly the PEG/cellulose film exhibited only one the lowest temperature zero order transition, which might be an indication of increased influence of the amount of PEG, where the amount of PEG present influences the degradation kinetic. The activation energy was the highest for PEG control, 312 kcal/mol. The activation energies for three transitions for cellulose and cellulose/PEG showed increasingly higher values for the transitions at higher temperatures (Figs. 2-4).

## Discussion

Bio-cellulose is proving to be a versatile and extremely varied as far as possible biomedical applications for this material. The wide spectra of possible applications include wound-healing and temporary wound coverage material; tissue engineered tendons and guided tissue regeneration. Bio cellulose was used as a physical barrier in the regeneration of periodontal tissue (17,18), where the separation allowed periodontal ligament cells and bone cells to proliferate within the wounded area leading to bone regeneration. Interestingly a pure material aspects and characterizations of the bacterial cellulose are sparse in the literature. In this study was have showed and important way of characterizing the bio cellulose with possible outcome of predicting the overall stability of the bio cellulose in various applications including dental research.
